# Infection Dynamics and Antimicrobial Resistance Profile of *Salmonella* Paratyphi B d-tartrate Positive (Java) in a Persistently Infected Broiler Barn

**DOI:** 10.3390/ijerph14010101

**Published:** 2017-01-21

**Authors:** Franziska Kloska, Martin Beyerbach, Günter Klein

**Affiliations:** 1 Veterinary Practice and Laboratory Wilms-Ellert-Klosterhalfen, Lohe 13, D-49429 Visbek, Germany; 2 Institute of Food Quality and Food Safety, University of Veterinary Medicine, Bischofsholer Damm 15, D-30173 Hannover, Germany; 3 Institute for Biometry, Epidemiology and Information Processing, University of Veterinary Medicine, Bünteweg 2, D-30559 Hannover, Germany; martin.beyerbach@tiho-hannover.de

**Keywords:** Infection dynamics, *S.* Java, broilers, antimicrobial resistance, veterinary public health

## Abstract

The infection dynamics of *S.* Java were examined in three consecutive rearing periods on a broiler farm in Northwestern Germany which had been persistently infected with *S.* Java for more than five years. The barn was investigated for *Salmonella* occurrence after cleaning and disinfection to verify the persistent contamination of the broiler house with *S*. Java before the start of the first rearing cycle. Confirmation of *Salmonella* absence in day-old chicks (time-point 1) as well as early establishment of infection between days 5–7 (time-point 2) were confirmed by caecal swabs prepared for qPCR and classical microbiological methods. At three time-periods (between days 11–15 (time-point 3), days 25–28 (time-point 4), and days 38–40 (time-point 5)) caecal content was examined for colony forming units (CFU) *Salmonella*/g. In general, there was an increase in *Salmonella* Java load at time-point 4 compared to time-points 3 and 5. Therefore, we observed a bell-shaped course of infection resulting in higher rates of *Salmonella* CFU/g prior to prethinning than at final slaughter. The antimicrobial susceptibility testing revealed resistance to tetracycline, fluorquinolones, trimethoprim, and cefoxitin.

## 1. Introduction

*Salmonella enterica* serovars are among the most important agents of food-borne outbreaks throughout the world. Poultry and poultry products are important sources for food-related salmonellosis in humans [1]. *Salmonella*, with its great diversity, is adapted to a variety of hosts. Environmental niches can be filled by several adapted serovars [2]. *S.* Java (correctly known as *S*. Paratyphi B d-tartrate positive) first appeared in poultry during the 1990s in the Netherlands and Germany, presumably introduced via feed or parent flocks [3].

Since then, there has been an increase in the prevalence of *S*. Java. In the EU in 2010, *S*. Java accounted for 4.6% of the serovars found in broiler meat. It became the third most prevalent serovar in the EU in 2010 due to high prevalences in the Netherlands (53.5%) and Germany (20.7%) [4]. Furthermore, non-European countries have also reported the incidence of *S*. Java in poultry (e.g., Saudi-Arabia [5] and Bangladesh [6]). In humans, this serovar leads to gastroenteritis and, to a minor degree, to invasive disease [7]. In the EU in 2014 it was the 11th most frequently reported serovar of human salmonellosis [8]. Outbreaks were described by Desenclos et al. [9] due to contaminated goat’s milk cheese or due to owning an aquarium along with a recent purchase of tropical fish or contaminated alfalfa sprouts [10,11]. Human infections in Germany and the Netherlands attributable to the consumption of poultry products were described by Toboldt et al. [12] and van Pelt et al. [13] as well as by Brown et al. in Scotland due to consumption of imported poultry [14]. It is a public health concern because of high occurrence rates of antimicrobial resistance, notably also to antimicrobials considered as critically important by joint experts of the Food and Agriculture Organization (FAO), the World Health Organization (WHO), and the World Animal Health Organization (OIE) (e.g., fluorquinolones or cephalosporines) [15,16,17].

Barns once infected with *S*. Java often become persistently infected with this serotype for the following rearing periods which is contrary to other serovars including *S*. Enteritidis or *S*. Typhimurium, as the author’s own observations in the poultry veterinary practice have shown. This is a potential risk factor for introducing *S*. Java into the downstream food processing plants and to the final consumer, but also to other farms via the same equipment or the same personnel employed for prethinning [18].

In 2004, van Immersel et al. [19] investigated the bacteria-host interaction of *S.* Java in vitro and in vivo in the poultry host. *S*. Java efficiently invades chicken epithelial cells and macrophages and can be found in caeca, the liver, and the spleen seven days post infection and up until slaughter, thus indicating its good adaptation to the poultry host.

This present study elucidates the infection dynamics and the antimicrobial resistance pattern of *S.* Java in a broiler flock under field conditions over three consecutive rearing periods. The acquired knowledge will help to better understand the bacteria-host interaction in the field and with it, benefits to develop intervention strategies as well as monitoring systems for the control of this serovar, thereby having an impact on public health.

## 2. Materials and Methods

### 2.1. Broiler Flocks and Farm Management

Three consecutive field surveys from June until November under commercial rearing conditions were carried out on a broiler farm in the northwestern part of Germany which had been persistently infected with *S*. Java for more than five years. Further information is given in Table 1 regarding flock size, breeding line, season, biosecurity, as well as vaccines and feed additives used.

### 2.2. Salmonella DNA Detection after Cleaning and Disinfection

To look for residual *Salmonella* organisms in the barn (see Table 2 for sampling procedure and results) samples were taken after routine cleaning and disinfection by an established risk-orientated hygiene analysis before the first rearing period either via boot swabs or by a bunch of surgical gauze swabs or pledgets moisturized with buffered peptone water (BPW, biomérieux, Nürtingen, Germany) [20]. Samples were tested by qPCR (see Section 2.4.) after incubation in BPW and Rappaport-Vassiliadis (RV) broth (Oxoid GmbH, Wesel, Germany). Biochemical and serological identification was performed by classical microbiology if qPCR was positive (see Section 2.5).

### 2.3. Salmonella Detection during Rearing Cycles

Broilers were tested for *Salmonella* occurrence at five different time-points during the rearing cycle: (1) shortly after arrival at the farm to ensure *Salmonella*-free chickens, (2) between days 5–7, (3) between days 11–15, (4) between days 25–28, (5) between days 38–40. At the first and second time points we took ten cloacal swabs (one swab/two animals) to see if a *Salmonella* infection was present at this early time-point. These were tested as pooled samples (five swabs as one pool) by qPCR after enrichment in BPW and RV broth (see Section 2.4 and Section 2.5). At the other time points we sampled caecal content of 20 randomly selected animals per time point. Broilers were stunned and killed according to § 4 and § 5 of German legislation for the protection of animal welfare on the farm [21] and transported to the laboratory within 15 minutes for further diagnostic proceedings in the course of veterinary routine supervision. Caecal content was taken under aseptic conditions in the laboratory and analysed to allow for *Salmonella* counting and to acquire quantitative data concerning *Salmonella* infection. We diluted the caecal content 1:10 in BPW and prepared two further 1:10 dilutions in maximum recovery solution (Oxoid GmbH, Wesel, Germany). One hundred microliters of each dilution were plated out onto brilliant green phenol red (BG) agar plates (Oxoid GmbH, Wesel, Germany) and incubated at 37 °C overnight (ca. 24 h). On the following day, we counted presumptive *Salmonella* colonies to determine *Salmonella* colony forming units (CFU) per g caecal content. The detection limit was 10^2^ CFU/g caecal content. As sometimes bacterial commensal flora was high, thus hampering microbiological diagnosis, each sample was tested in qPCR, allowing us to obtain a qualitative result. For qPCR, Malorny et al. mention a detection limit of 3 CFU/g after enrichment in the presence of background flora [22]. Positive samples were further analysed biochemically and serologically (see Section 2.5). The first dilution in BPW was incubated at 37 °C overnight. Afterwards, 100 µL were transferred to RV broth for selective enrichment (42 °C overnight incubation) and for use in qPCR for *Salmonella* DNA confirmation.

### 2.4. Quantitative PCR (qPCR) Set-up and Amplification

In our study, we used a commercial testkit for DNA-extraction and 5’ nuclease real-time PCR (Kylt^TM^, AniCon GmbH, Höltinghausen, Germany). *Salmonella* qPCR was first published by Malorny et al. [22]. One millilitre of the RV-broth was used for the extraction-purification step and retention of bacterial DNA. The reaction mix contained Taq-polymerase and primers ttr-6 (CTCACCAGGAGATTACAACATGG) as well as ttr-4 (AGCTCAGACCAAAAGTGACCATC). PCR was performed in an ep realplex thermocycler (Eppendorf AG, Hamburg, Germany). The PCR reaction conditions were as follows: 95 °C for 1 min as a primary denaturation step followed by 45 cycles of 95° C for 15 s and 65° C for 60 s. The mean and standard deviation (SD) of the signal of all probes in cycles 3–10 were calculated and the threshold was set at mean +(10 × SD).

### 2.5. Biochemical and Serological Identification of Salmonella

One hundred microliters of RV broth of qPCR positive samples were plated out onto BG agar plates and incubated at 37 °C for 24 ± 2 h. Three suspicious colonies were selected, subcultivated, and biochemically identified (api^®^20E, biomérieux, Nürtingen, Germany). Isolates identified as *Salmonella* were serotyped with *Salmonella* antisera (sifin diagnostics GmbH, Berlin, Germany). Fermentation of d-tartrate was tested by overnight inoculation of the bacteria in Phenol Red tartrate agar (15 g agar, 10 g peptone, 10 g potassium tartrate, 5 g NaCl, 0.024 g Phenol Red/l water; pH 7.6).

### 2.6. Antimicrobial Susceptibility Testing of S. Java

We tested one isolate per survey using the agar diffusion test as a screening step with antibiotic disks (Oxoid GmbH, Wesel, Germany) for antimicrobial resistance to ampicillin (10 µg AMP), spectinomycin (10 µg SPC), streptomycin (25 µg STM), and sulfamethoxazole/trimethoprim (23,75/1,25 µg SXT) in accordance with DIN 58940-3: medical microbiology—susceptibility testing of pathogens to antimicrobial agents [23]. Furthermore, the tested isolates were sent to the German National Reference Laboratory for Antimicrobial Resistance located at the German Federal Institute for Risk Assessment (BfR, Berlin). Testing to determine resistance to ampicillin (AMP), ceftazidin (TAZ), chloramphenicol (CHL), ciprofloxacin (CIP), tetracycline (TET), tigecycline (TGL), gentamycin (GEN), trimethoprim (TMP), colistin (COL), cefoxitin (FOX), cefotaxim (FOT), cefepim (FEP), nalidixic acid (NAL), and sulfmethoxazole (SMX) was performed by microdilution in accordance with Clinical & Laboratory Standards Institute guideline M31_43/2006. Epidemiological cut-off values (ECOFF) of the European Committee on Antimicrobial Susceptibility Testing (Eucast) were used for interpreting resistance (ww.eucast.org) [24].

### 2.7. Statistical Analysis

Data were analysed with SAS^®^ software, version 9.3 (SAS Institute Inc., Cary, NC, USA). One-way and two-way analyses of variance were used to analyse the amount of *S*. Java CFU/g at time-points 3, 4, and 5 by the least significant difference test (LSD-test) regarding the pairwise differences of the means. This test holds the comparisonwise error rate of 0.05. Culturally negative but PCR positive samples were counted as 5 × 10^1^ CFU/g. CFU was log-transformed (log CFU/g) to generate a normal distribution of the residuals. Due to antibiotic treatment prior to sampling, rearing period one was regarded alone in the quantitative analysis of field surveys. The amount of positive samples by direct microbiology in surveys two and three or by qPCR for all three rearing periods were compared at the time-points using Pearson’s chi-square test for homogeneity or Fisher’s exact test. Logistic regression and Odds Ratios (ORs) were calculated to ascertain the effect of time on culturally or qPCR positive samples. *P*-values < 0.05 were considered statistically significant.

## 3. Results

### 3.1. Salmonella DNA Detection after Cleaning and Disinfection

After cleaning and disinfection we could demonstrate the persistent occurrence of *S*. Java in the barn at different tested control points (see Table 2). Seven out of fifteen control points were positive for *S*. Java, showing a high level of contamination.

### 3.2. Salmonella Detection during Rearing Cycles

**Figure 1:**
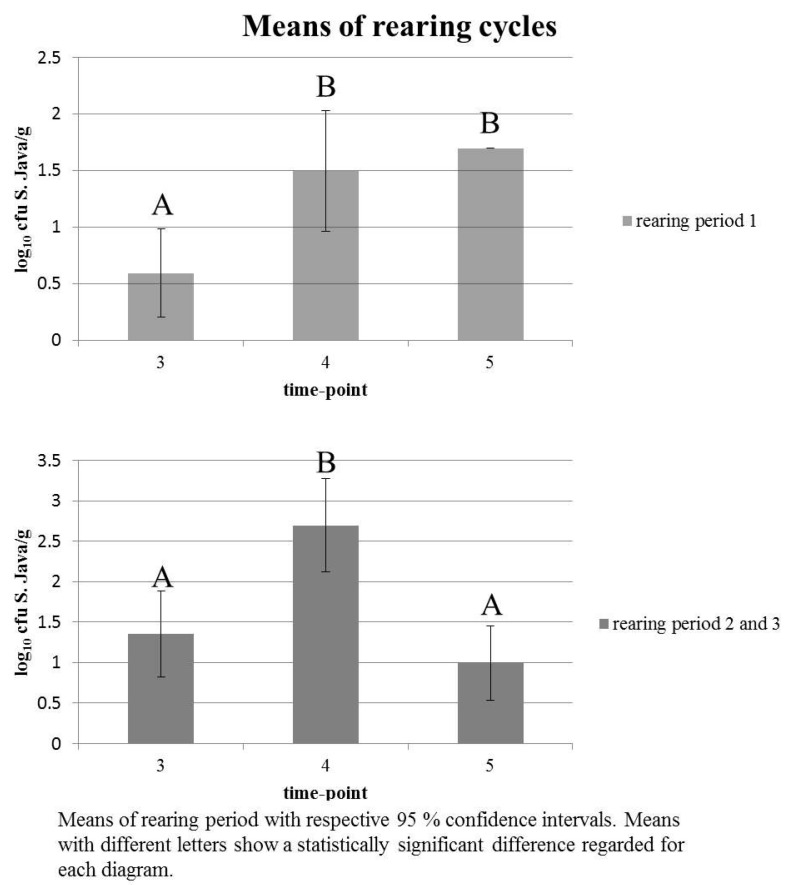
Means of the rearing periods at the respective sampling time-point.

Day-old chicks were *S.* Java negative. The early establishment of *S.* Java infection at time-point 2 was confirmed. Overall, the wide 95% confidence interval (CI) at time-points 3, 4, and 5 suggests a heterogeneous colonisation of *S.* Java in broilers. In general, there was a significant difference between time-points 3 and 4 as well as 4 and 5, respectively, but not between time-points 3 and 5 (see Table 3 and Figure 1). When antibiotic treatment occurred, the result was different (see Table 4 and Figure 1), showing a low, but homogeneous colonisation of chickens with *S*. Java shortly after the end of therapy with no significant difference of colonisation at time-points 4 and 5, but only at time-points 3 and 4. After therapy, positive results were only obtained for qPCR, not for direct microbiology.

After dichotomisation of data of surveys two and three (*S.* Java positive by direct plating—yes or no) Fisher’s exact test showed dependency of culturally positive results compared to the time-point tested. Time-point 4 significantly resulted in more positive samples than time-point 3 (*p* < 0.0001). The Odds Ratio of positive results at time-point 4 versus (vs.) time-point 3 amounted to 3.333 (95% CI 1.420–7.823). The pairwise comparison of time-point 5 vs. 3 showed that the Odds Ratio was 0.455 (95% CI 0.145–1.421) with no significant difference between time-points (*p* > 0.05). The Odds Ratio of time-point 5 versus time-point 4 was 0.136 (95% CI 0.048–0.390, *p* < 0.001). Similar results were obtained for qPCR positive samples comparing three rearing cycles (see Table 5). Again, time-point 4 differed significantly from time-points 3 and 5 (*p* < 0.05).

### 3.3. Biochemical and Serological Identification of Salmonella

Biochemical and serological testing revealed *Salmonella* enterica serovar Paratyphi B d-tartrate positive (1,4,12:b:(1,2)) in all cases. All isolates were O:5 negative.

### 3.4. Antimicrobial Susceptibility Testing of S. Java

In the agar diffusion test, *S.* Java showed resistance to spectinomycin, streptomycin, and was intermediate for ampicillin. In the microdilution test the isolates were resistant to tetracycline, nalidixic acid, ciprofloxacin, and cefoxitin. *S*. Java was found to be resistant to sulfamethoxazole/trimethoprim in the agar diffusion test, as well as to trimethoprim in the microdilution test. For sulfamethoxazole in the microdilution test, the three tested isolates indicated inconsistent minimal inhibitory values ranging from 16µg/mL, 32µg/mL, to 64 µg/mL.

## 4. Discussion

The data indicate a great variation per time-point in the load of *S.* Java in the caecal content of broilers sampled within a persistently infected flock as shown in the great range of 95% confidence intervals. This results in a heterogeneous colonisation of chickens with *S.* Java regarded per time-point, except in the case of antibiotic treatment prior to sampling (compare results of time-point 5 in rearing period one when there is no range of 95% confidence interval). The data suggest a peak of colonisation at time-point 4 compared to time-points 3 and 5, both confirmed by direct plating and qPCR. Chickens have a greater chance of being positive at time-point 4 which results in more *S.* Java positive chickens slaughtered at prethinning (around day 30) than at 40 days of age. This poses a greater risk of contamination of the slaughtering line with *S*. Java. If we further consider that chickens slaughtered at prethinning are intended to be merchandised unprocessed, this might additionally increase the exposure of the final consumer. Furthermore, during prethinning, equipment and personnel are brought to the farm, thus posing a risk of additional *S*. Java contamination on this farm as well as on other farms, as equipment and personnel are often employed on more than one farm [18]. The variation in the individual *Salmonella* status of broilers should be considered when samples are taken at slaughter for risk assessment, as was previously shown by Hansson et al. [25] for *Campylobacter* spp.

Van Immerseel et al. [19] demonstrated that one week after experimental infection all chickens were positive for *S.* Java, this being confirmed with cloacal swabs directly plated onto BG agar. Animals infected by a seeder model were all positive after two weeks post infection, this also being confirmed by cloacal swabs that were directly plated onto BG agar. At slaughter age (six weeks) all caecal samples were positive on BG agar, but only after enrichment. These results are in accordance with our data which also suggest a decrease in positive results by direct plating the older the animals become.

Day-old chicks are always free of *S*. Java. It is therefore of utmost importance to house chickens in a *S*. Java-free environment. The studied barn was cleaned and disinfected according to standard protocols which were insufficient to eradicate *S*. Java. Therefore, there is always a risk of newly housed chickens becoming infected. It is well known that *S.* Java persists in broiler houses for a long time and is therefore the source of infection of day-old chicks in the following rearing period. The data in Table 2 indicate that the studied broiler house was still contaminated with *S.* Java after cleaning and disinfection before arrival of the new birds. The fact that 7 out of 15 sampling points were positive for *S*. Java indicates a high level of contamination, although the level of contamination might vary between different rearing periods. We demonstrate in another paper that only routine cleaning and disinfection alone would not be appropriate for eradication of *S*. Java from the farm [20]. At least two disinfection steps were required for successful eradication of *S*. Java on the farm. We implemented a feasible risk-orientated hygiene analysis examining individual critical control points of the barn for successful eradication of the pathogen. New chickens were not housed before all sampling points had been tested negative in qPCR for *Salmonella* occurrence. Prethinning, as a risk factor for reintroduction of *S*. Java, was abolished.

The studied farm has been positive for *S*. Java for more than five years. For that reason it is more than likely that the birds in the following rearing period become *Salmonella* positive. The detected differences in the caecal colonisation of the birds in the different rearing periods might be due to the different levels of contamination in the broiler houses when the birds arrived at the farm. On the other hand, young chickens are very susceptible to infection with *Salmonella* in general. At two weeks of age, when we first performed a quantitative examination of *S*. Java, incidences of *S*. Java might be high due to the excretion of *S*. Java by birds being first infected due to contamination of the environment. This fact results in further infection of other birds, comparable to a seeder model. The detailed quantitative level of contamination in the houses was not tested as the extra costs are not justifiable for routine testing. Presently, the most important aim to further reduce the contamination of poultry products with *Salmonella* organisms, and therefore to reduce the risk for humans, is to transfer day-old broilers to a *Salmonella*-free environment, as in most cases newly hatched chicks are free of *Salmonella* organisms. This demand is absolutely essential for further control of *Salmonella* spp. in poultry.

During rearing period one, animals received a combined antimicrobial therapy with amoxicillin and colistin which was stopped three days prior to sampling. The therapy seemed to result in a uniform colonisation of chickens with *S.* Java (compare Table 4 and Figure 1) at a low level, but was not capable of eliminating the pathogen from the population. Antimicrobial therapy can reduce the transmission and excretion of *S.* Java, perhaps below the level of detection, but sustains the persistence of *S*. Java. This might even result in a reduced diagnostic sensitivity of microbiological methods without enrichment used in this study when samples are taken after antimicrobial treatment and might therefore interfere with the diagnosis of *S*. Java. Antimicrobial treatment is no alternative to good hygienic practices and biosecurity measures that might lead to the elimination of *S.* Java. Antimicrobial treatment of broilers also poses a risk of carcass contamination with resistant *S.* Java as well as resistant commensal bacteria [26].

After antimicrobial treatment and subsequent sampling in trial 1, positive results were only obtained with qPCR as there had been no detection of bacterial growth on BG agar. On the other hand, there might have been a high level of competing commensal flora that may complicate microbiological diagnosis. Therefore, we used qPCR to obtain a qualitative result concerning the *S*. Java status of animals. For qPCR, it has been proven by using artificial culture mixes and after enrichment that background microorganisms have no influence on the detection limit (3 CFU/mL) of *Salmonella* organisms [22].

During recent years there has been a rise in awareness concerning the selection of antimicrobial resistance among food-producing animals and its potential risk concerning public health [27]. Between 1960 and 1993 there was a great diversity among isolates of *S.* Java. However, since 1994 multidrug-resistance and the predominance of one single clone have been obvious [28]. Strains of this group emerged due to the increased use of antimicrobials to manage the *S.* Enteritidis crisis at the beginning of the 1990s. They have no integron class 1, but multidrug-resistance is due to the acquisition of class-2 integrons in the bacterial chromosome. The core spectrum of resistance is directed against trimethoprim, spectinomycin, and streptomycin and is mediated by the gene cassette dfrA1-sat1-aadA1 carried by transposon Tn*7* [16]. This gene cassette also seems to be expressed by the studied isolates shown by antimicrobial susceptibility testing but needs further confirmation by molecular methods. Occurrence of *Bla*_TEM_-genes in *S.* Java have been described by Hasman et al. [29] in Dutch poultry and meat thereof as well as occurrence of *Bla*_CTX_-genes and *Bla*_TEM_-genes by Rodriguez et al. [30] in ceftiofur-resistant *Salmonella* enterica isolates from chickens and chicken meat in Germany. They state that extended spectrum β-lactamase (ESBL) activity as well as AmpC-β-lactamase activity increase in *S.* Java due to a variety of genetic mobile elements capable of horizontal gene transfer leading to a new onset of a public health problem. Isolates investigated in Belgium from 2008–2010 showed a plasmid-borne resistance towards ESBL antimicrobials. Even though these antimicrobials are not indicated for use in poultry there was a resistance selection as plasmids conferring ESBL resistance often have other resistance genes (e.g., against sulphonamides or trimethoprim) and these antimicrobials are frequently used in the veterinary poultry sector [17]. Van Pelt et al. [13] found that *S*. Java becomes less sensitive to ciprofloxacin, this also being confirmed in our study. It is important for the therapy of invasive *Salmonella* infections in adults [31]. The interpretation of occurring antibiotic resistance is somewhat difficult as ECOFFs are not always established for *S.* Java. For example, the minimum inhibitory concentration for cefotaxime is exactly the same as the published ECOFF for *Salmonella* spp. It is a sensitive substrate for confirming CTX-M enzymes, which are the most common ESBL enzymes. On the other hand, cefoxitin is a sensitive, but less specific parameter for confirming AmpC-producing enterobacteriaceae [27]. One point to consider is the intermediate resistance profile of *S.* Java to ampicillin in the agar diffusion test that was not confirmed in the microdilution test, but might be further evidence for the expression of AmpC enzymes. Thus, additional molecular tests for the presence of ESBL and/or AmpC enzymes are needed to affirm their occurrence in the studied isolates. Resistance profiles might be misinterpreted because of missing established ECOFFs for *S*. Java or varied results in the agar diffusion and microdilution, test as is the case with sulfamethoxazole/trimethoprim in this study. Further molecular methods and epidemiological data for interpreting and establishing ECOFFs are required for precise interpretation of the resistance profile of the studied isolate. In the agar diffusion test, *S*. Java was found to be resistant to sulfamethoxazole/trimethoprim as well as to trimethoprim in the microdilution test. For sulfamethoxazole, the three tested isolates ranged from minimal inhibitory concentrations of 16µg/mL, 32µg/mL, and 64µg/mL. No ECOFF for S. Java and sulfamethoxazole has been established so far, making interpretation of results difficult. Schroeter et al. [32] used a breakpoint of 512 µg/mL to assess resistance of *Salmonella* species to sulfamethoxazole in their study. In this case, the studied isolate was not resistant to sulfamethoxazole. Additionally, resistance to sulfamethoxazole is mediated by *Salmonella* Genomic Island I (SGI I) [33]. *S.* Java comprises two different lineages that either express the O:5 antigen or do not [34]. O:5 positive strains possess further virulence genes that allow it to interact with a greater range of hosts, whilst O:5 negative strains have the ability to persist in poultry in the Netherlands, Germany, and Belgium and possess less virulence, but are multiresistant. O:5 positive strains express SGI I that usually confers resistance to sulfamethoxazole. The studied isolates seem to belong to the group of O:5 negative less invasive strains which persist in poultry and exhibit a range of antimicrobial resistance, but do not express SGI I [34].

Another serovar persistent in poultry in France, *S*. Senftenberg, shows a low capacity to invade chicken enterocytes and to pass the intestinal wall [35]. In contrast, *S*. Java invades chicken enterocytes and macrophages. It can be found in caeca, the liver, and the spleen of infected broilers and is able to spread efficiently within chickens [19]. Nonetheless, the invasiveness of *Salmonella* and its virulence are not always correlated [36]. The ability to colonise a particular host is diverse and involves the host (e.g., age and breeding differences), the serovar, and extrinsic pressures (e.g., antimicrobial therapy, stress, coccidiosis), and this interplay leads to the observed variety in host ranges observed in different countries [37,38].

## 5. Conclusions

In conclusion, the presented study contributes to a better understanding of the infection dynamics of *S.* Java in broilers under field rearing conditions. Animals slaughtered at prethinning have a higher risk of being *S*. Java positive, thus resulting in a higher exposure of the final consumer. Infection with *S*. Java results in a heterogeneous colonisation of chickens in general. A low, but homogenous colonisation of CFU S. Java/g caecal content can be found after antimicrobial therapy. Both points must be considered when samples are taken for risk assessment (e.g. at slaughterhouses). The presented data underline the importance of implementing good surveillance programmes and the development as well as establishment of effective control measures (e.g., intensive cleaning and disinfection methods attended by crucial testing of critical control points for *S*. Java occurrence). Of great importance is the placement of day-old chickens in a *Salmonella*-free environment. A contaminated housing generally is the infection source in the case of *S.* Java infection. The antimicrobial multiresistance of *S.* Java can jeopardise public health. Further monitoring of antimicrobial resistance of animal and human isolates as well as the establishment of ECOFFs for *S.* Java are both required for assessing the public health risk related to *S*. Java.

## Figures and Tables

**Table 1:** Rearing conditions during field surveys: detailed conditions used in the three field surveys including flock information, biosecurity, feed additives used, and antibiotic therapy.

Type of Condition	Field Survey 1	Field Survey 2	Field Survey 3
flock information			
flock size	33,000	33,000	33,000
breed	Ross 308	Ross 308	Ross 308
season	June–August	August–September	October–November
biosecurity			
rubber boots	changed	Changed	changed
clothes	not changed	not changed	not changed
prethinning	yes	Yes	yes
vaccination (first 15 days of life)	Gumboro disease, Newcastle disease, Infectious bronchitis	Gumboro disease, Newcastle disease, Infectious bronchitis	Gumboro disease, Newcastle disease, Infectious bronchitis
feed additives			
first 18 days of life	vitamins A, D_3_, E ^1^	vitamins A, D_3_, E ^1^	vitamins A, D_3_, E ^1^
whole rearing period	buffered acids *	buffered acids *	buffered acids *
antibiotic therapy	amoxicillin/colistin on days 33–35	lincomycin-spectinomycin on days 1–4	lincomycin-spectinomycin on days 1–4

^1^ applied via the drinking water several times over two to three days; * a commercially available product, applied via the drinking water repeatedly during the whole rearing period, not applied during antimicrobial treatment and vaccination.

**Table 2:** Sampling regime and qPCR-results: *Salmonella* presence in the barn after cleaning and disinfection was confirmed with the following sampling plan before the start of the first rearing period. All qPCR positive samples were confirmed as *S*. Java by classical microbiology.

Sampling Point	qPCR Result
water lines	negative
feed lines	negative
feed suppliers	negative
Wall	positive
supply air system	positive
exhaust air system	positive
barn gateway	positive
heating system	negative
windows and window frames	negative
Ceiling	negative
cable duct at the ceiling	positive
ligament suspension of lines	negative
floor, joints, and crannies	positive
anteroom	positive
housing forecourt	negative

**Table 3:** Descriptive statistics for rearing period two and three with the respective mean, minimum, and maximum, as well as 95% confidence interval (CI) presented as log CFU/g caecal content at each sampling point for quantitative analysis and the result of the least significant difference (LSD) test.

Time Point			Statistic
**3**	**4**	**5**	
1.349	2.694	0.990	mean
0	0	0	min
5.322	5.602	5.204	max
0.818; 1.880	2.118; 3.270	0.528; 1.451	95% CI
A *	B *	A *	LSD test

* Means with different letters show a statistically significant difference.

**Table 4:** Descriptive statistics for rearing period one with the respective mean, minimum, and maximum, as well as 95% confidence interval (CI) presented as log CFU/g caecal content at each sampling point for quantitative analysis and the result of the least significant difference (LSD) test.

Time Point			Statistic
**3**	**4**	**5 ^1^**	
0.594	1.497	1.698	mean
0	0	1.698	min
1.698	4.954	1.698	max
0.205; 0.983	0.962; 2.031	-	95% CI
A *	B *	B *	LSD test

^1^ Please note the antimicrobial treatment stopped three days prior to sampling; * Means with different letters show a statistically significant difference.

**Table 5:** qPCR positive and negative samples depending on the tested time-point: It shows the qPCR results of three rearing periods indicating significant differences of time-point 4 compared with time-points 3 and 5.

**Time-Point**	**Positive**	**Negative**	***p*-Value**
**3**	34	26	
**4**	12	48	
**5**	24	36	
**Time-Point**	**Odds Ratio**	**95% CI**	
**4 vs. 3**	5.231	2.320; 11.793	<0.0001
**5 vs. 3**	1.962	0.949; 4.055	>0.05
**5 vs. 4**	0.375	0.166; 0.849	<0.05
